# Analysis of hepatic fibrosis markers in the serum of chronic hepatitis B patients according to basal core promoter/precore mutants

**DOI:** 10.1038/s41598-022-14285-9

**Published:** 2022-06-17

**Authors:** Caroline Lefeuvre, Marine Roux, Simon Blanchard, Hélène Le Guillou-Guillemette, Jérôme Boursier, Françoise Lunel-Fabiani, Pascale Jeannin, Adeline Pivert, Alexandra Ducancelle

**Affiliations:** 1 Univ Angers, CHU Angers, HIFIH, SFR ICAT, F-49000 Angers, France; 2 Univ Angers, HIFIH, SFR ICAT, F-49000 Angers, France; 3 Univ Angers, INSERM Unité 892, CNRS Unit 6299, F-49000 Angers, France

**Keywords:** Hepatitis, Hepatitis, Liver diseases, Virology, Hepatitis B virus, Viral pathogenesis, Cytokines, Microbiology

## Abstract

The A1762T/G1764A double mutant in the basal core promoter (BCP) region of the hepatitis B virus (HBV) is associated with severe hepatic lesions while the G1899A mutation with the double mutant is associated with a significant reduction in the risk of severe fibrosis. This study aims to measure a number of markers in the serum of patients with chronic HBV infection and to assess relationships between these markers and BCP/precore mutants with consideration of the stage of fibrosis. The serum levels of resistin, TGF-β1, MMP-1, TIMP-1, collagen IA1 and PDGF-BB, which are markers that are known to be involved in the process of hepatic fibrosis, were assayed. The serum levels of PDGF-BB and TIMP-1, and the mutation profile were independently associated with advanced fibrosis. A higher level of TIMP-1 was associated with advanced fibrosis regardless of the mutation status, and a higher level of PDGF-BB was associated with nonsevere fibrosis in patients infected with viruses harboring the A1762T/G1764A or A1762T/G1764A/G1899A mutations. Our results suggest an impact of the A1762T/G1764A mutant on the biological pathway related to TGF-β1 and PDGF-BB. In vitro studies are needed to understand the impact of these mutants on the serum secretion of markers involved in fibrosis severity.

## Introduction

At present, chronic hepatitis B (CHB) remains a serious global health concern, with approximately 296 million people living with CHB infection worldwide^1^. Given that the virus is not directly cytopathic in itself, it is the conflict intensity between the hepatitis B virus (HBV) and the host immune response^2,3^ that determines the control of HBV infection, the development of liver damage (fibrosis, cirrhosis or hepatocellular carcinoma [HCC]) and the progressive selection of HBV variants. The typical course of HBV infection tends to involve a hepatitis B e antigen (HBeAg)-positive phase with high HBV DNA replication. This is followed by an HBeAg seroconversion phase with clinical remission and a decline in HBV viral load^4^. Nevertheless, during the HBeAg seroconversion phase, immune pressure can select a number of genetic variants in the basal core promoter (BCP) and precore (PC) regions that downregulate or abolish HBeAg production with active DNA replication^5,6^. Chronic HBeAg-negative hepatitis B has become the predominant type of CHB infection in France^7^ and worldwide. However, the impact of these mutations on the natural course of infection and on the severity of liver damage has not yet been clearly established for some of these mutants. The G1896A mutation in codon 28 of the PC region is the most frequently described mutation in this region and creates a premature stop codon that stops HBeAg synthesis^8^. Some studies reported a relationship between the G1896A mutation and severe liver hepatitis, whereas others reported no associations between the mutation and clinical outcomes^9,10^. In the BCP region, the A1762T/G1764A double mutant has been found to downregulate precore mRNA production, resulting in reduced HBeAg secretion^11^. An association between the A1762T/G1764A double mutant and severe hepatic lesions or increased risk for HCC has been frequently described^12–16^. We recently demonstrated in a national multicenter clinical study that there was no significant correlation between the G1896A mutation and severe fibrosis, and that the simultaneous presence of the G1899A mutation (in the PC region) with the A1762T/G1764A double mutant significantly reduced the risk of severe fibrosis compared to the presence of the double mutant alone. The G1899A mutation seems to be an independent protective factor against severe fibrosis^17^. The impact of these mutants remains to be elucidated.

Hepatic fibrosis is the result of chronic tissue injury and inflammation caused by various factors such as alcohol consumption, viral hepatitis and nonalcoholic steatohepatitis^18^. Fibrosis is a dynamic and reversible process that is characterized by quantitative and qualitative changes in the extracellular matrix (ECM). Excessive and persistent accumulation of the ECM can progressively lead to the destruction of normal liver architecture with impairment of liver microcirculation and liver cell functions, and result in the development cirrhosis^19^. Immune cells, in particular human hepatic stellate cells (HSCs), and the relevant cytokines are closely related to the pathogenesis of hepatitis B.

Among the factors involved in the fibrosis process, we can distinguish the products of ECM synthesis and degradation (collagens) by the proteases that regulate their production or modification, such as matrix metalloproteinases (MMPs), their inhibitors (TIMPs) and fibrogenesis-related regulatory factors such as transforming growth factor beta type 1 (TGF-β1) or platelet derived growth factor-BB (PDGF-BB). Previous studies have investigated markers (TGF- β1, resistin and MMP-1) associated with fibrosis severity in the serum of infected patients according to the stage of hepatitis B disease (CHB, liver cirrhosis or liver failure)^20–22^. However, to our knowledge, no studies to date have investigated the secretion of these types of markers in serum by taking into consideration the presence or absence of the BCP/PC mutants that are described to have an impact on the fibrosis process.

Our study assayed the serum levels of resistin, TGF-β1, MMP-1, TIMP-1, collagen IA1 (Col IA1) and PDGF-BB. These are markers that are considered to be associated with the liver fibrosis process, but they are not currently used routinely as predictive markers for liver fibrosis. However, our aim is not to determine the most predictive marker in liver fibrosis. We analyzed the serum levels of these markers in patients with chronic HBV infection according to the fibrosis stage (METAVIR score) and the profile of mutational changes in the BCP/PC region (especially at nucleotide positions 1762/1764/1899).

## Results

### Characteristics of patients

Table 1 shows the baseline characteristics of patients, both globally and according to liver fibrosis stage (< F3 and ≥ F3). Both METAVIR liver fibrosis stage and BCP/PC mutation status were available for 79 patients. Among the 79 patients, 27 had advanced fibrosis (≥ F3) and 52 had nonsevere fibrosis (< F3). Patients with severe fibrosis (≥ F3) were significantly older (p value < 0.001) and had higher ALT levels (p value = 0.010) than patients in the nonsevere fibrosis group. The severity of liver fibrosis was dependent on the mutation profile in the BCP/PC region (p value = 0.013). Supplementary Table S1 (see Supplementary data) summarizes the HBV viral load data and the presence/absence of HBeAg according to fibrosis stage and mutation profile. Supplementary Table S2 (see Supplementary data) summarizes the distribution of the A1762T/G1764A double mutant and A1762T/G1764A/G1899A mutations and severe/nonsevere fibrosis stage according to HBV genotypes.

**Table 1:** Baseline characteristics of the enrolled patients (according to METAVIR liver fibrosis stage [< F3 vs. ≥ F3]).

	All patients (n = 79)	< F3 (n = 52)	≥ F3 (n = 27)	p
**Age (years)**	39.6 ± 16.2	33.9 ± 13.7	50.6 ± 15.2	< 0.001
**Sex**				
Male	47 (59.5%)	27 (51.9%)	20 (74.1%)	0.097
Female	32 (40.5%)	25 (48.1%)	7 (25.9%)	
**HBV genotype**				
A	36 (46.7%)	29 (56.9%)	7 (26.9%)	–
B	2 (2.6%)	1 (2.0%)	1 (3.9%)	
C	9 (11.7%)	4 (7.8%)	5 (19.2%)	
D	13 (16.9%)	8 (15.7%)	5 (19.2%)	
E	12 (15.6%)	5 (9.8%)	7 (26.9%)	
F	4 (5.2%)	4 (7.8%)	–	
G	1 (1.3%)	–	1 (3.9%)	
Missing data	2	1	1	
**BCP/PC mutation status**				
Wild type	22 (27.8%)	19 (36.5%)	3 (11.1%)	0.013
A1762T/G1764A	41 (51.9%)	21 (40.4%)	20 (74.1%)	
A1762T/G1764A + G1899A	16 (20.3%)	12 (23.1%)	4 (14.8%)	
**HBV DNA (log IU/mL)**	5.0 [3.3; 7.1]	4.1 [3.3; 6.1]	5.7 [4.7; 7.2]	0.094
Missing data	2	1	1	
**HBeAg**				
Negative	48 (68.6%)	34 (73.9%)	14 (58.3%)	0.288
Positive	22 (31.4%)	12 (26.1%)	10 (41.7%)	
Missing data	9	6	3	
**Anti-HBe Ab**				
Negative	22 (28.2%)	12 (23.1%)	10 (38.5%)	0.248
Positive	56 (71.8%)	40 (76.9%)	16 (61.5%)	
Missing data	1		1	
**Elevated ALT**				
No	43 (55.1%)	34 (66.7%)	9 (33.3%)	0.010
Yes	35 (44.9%)	17 (33.3%)	18 (66.7%)	
Missing data	1	1		

*< F3* nonsevere fibrosis (F0–F2), *≥ F3* severe fibrosis (F3–F4), *HBV* hepatitis B virus, *BCP* basal core promoter, *PC* precore, *HBeAg* hepatitis B e antigen, *Ab* antibody, *ALT* alanine aminotransferase.

### Univariate analyses: marker serum levels according to liver fibrosis stage or BCP/PC mutants

#### Marker serum levels according to liver fibrosis stage

**Figure 1:**
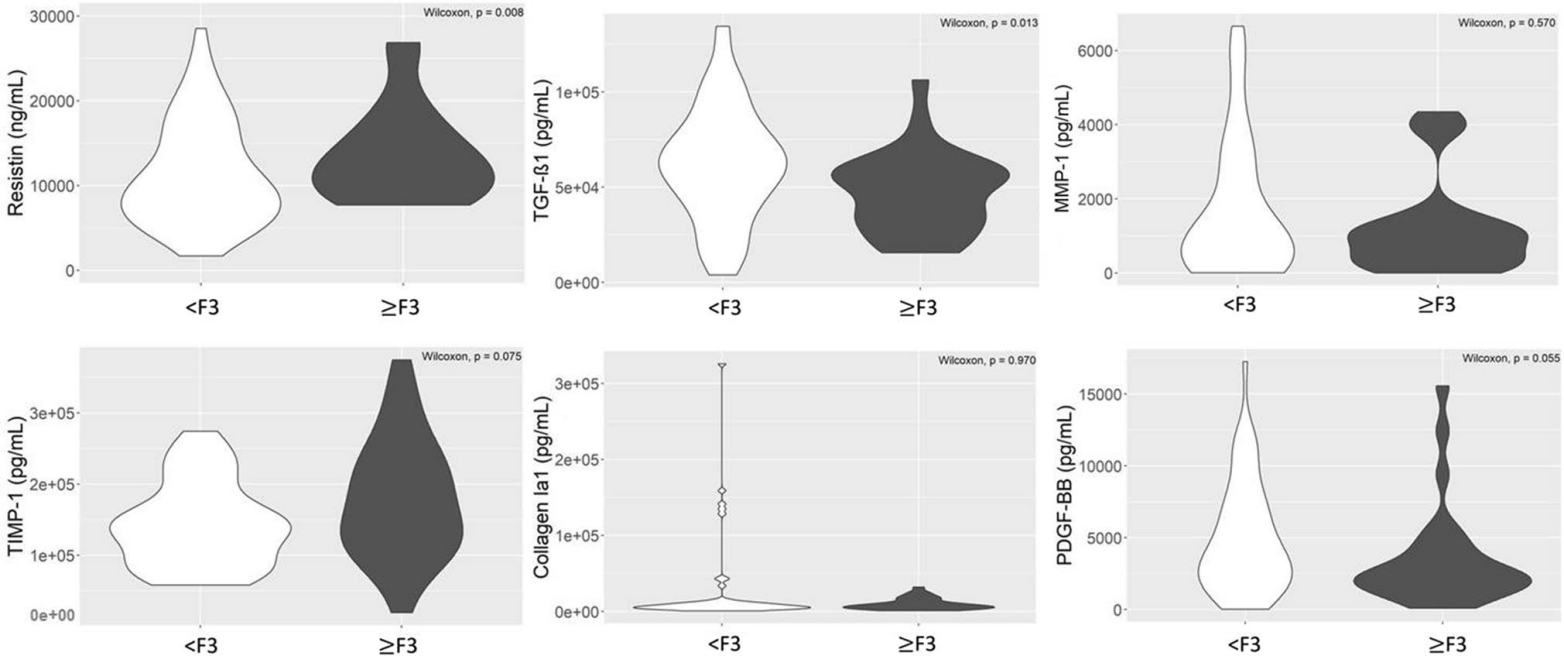
Distribution of fibrosis markers (resistin, TGF-β1, MMP-1, TIMP-1, collagen IA1 and PDGF-BB) according to fibrosis stage (< F3; ≥ F3). A p value lower than 0.05 was considered statistically significant.

The distribution of all marker serum levels according to liver fibrosis stage is summarized in Fig. 1. High serum resistin levels were detected in patients with severe fibrosis stage (≥ F3) (p value = 0.008). In contrast, high serum TGF-β1 and PDGF-BB levels were measured in patients with nonsevere fibrosis (p value = 0.013 and p value = 0.055, respectively).

The serum levels of MMP-1 and collagen IA1 were not significantly different between severe and nonsevere fibrosis stages (p value = 0.570 and p value = 0.970, respectively). While the serum level of TIMP-1 was not significantly different according to severe and nonsevere fibrosis stages (p value = 0.075), we observed a tendency for this to be higher in the severe fibrosis group (Fig. 1).

#### Marker serum levels according to BCP/PC mutants

**Figure 2:**
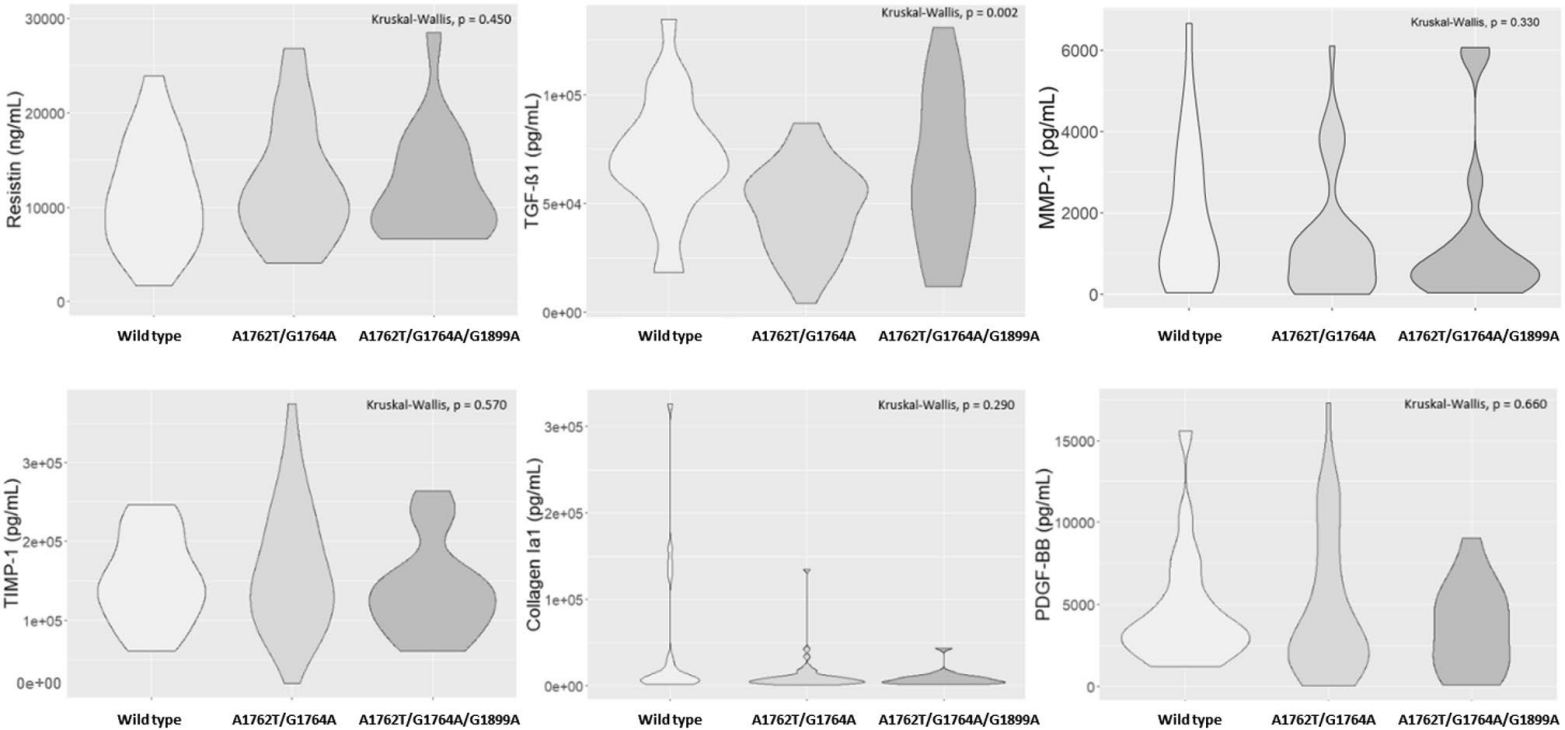
Distribution of fibrosis markers (resistin, TGF-β1, MMP-1, TIMP-1, collagen IA1 and PDGF-BB) according to the BCP/PC mutants (wild type; A1762T/G1764A; A1762T/G1764A/G1899A). A p value lower than 0.05 was considered statistically significant.

The distribution of all marker serum levels according to BCP/PC mutation is summarized in Fig. 2. The serum levels of TGF-β1 were significantly differently distributed according to the mutation profile (p value = 0.002). More precisely, a significantly higher serum level was observed for the wild type compared to the A1762T/G1764A mutant (p value < 0.001). We did not observe a significant difference in the distribution for the serum levels of the other markers according to the mutation profiles (p values > 0.05) (Fig. 2).

### Multivariate analyses: marker serum levels according to liver fibrosis stage and BCP/PC mutants

#### Exploratory step

**Figure 3:**
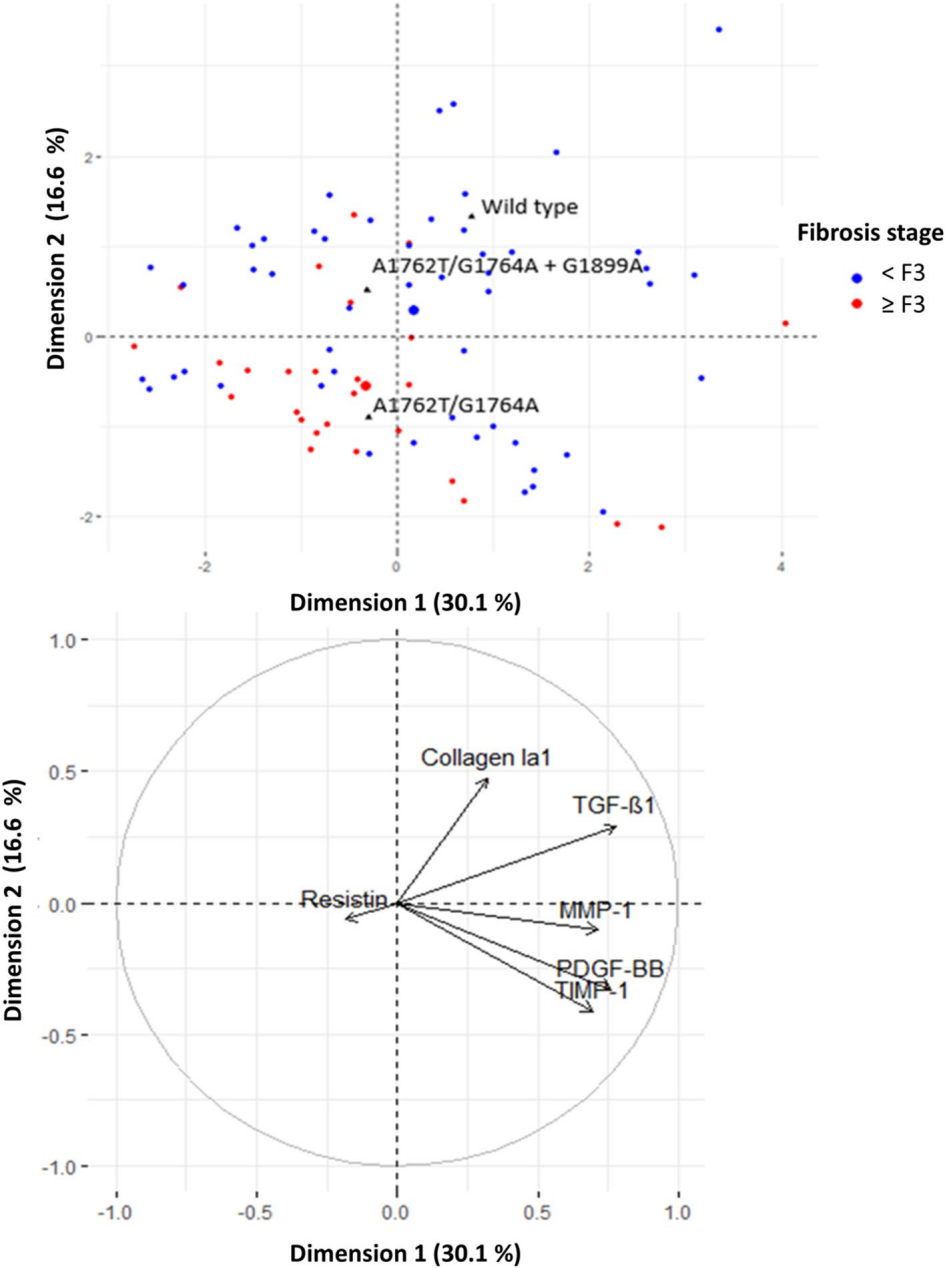
Factor Analysis of Mixed Data (FAMD) (categorical data [top] and continuous data [bottom]). Individuals with nonsevere fibrosis are plotted in blue, and individuals with advanced fibrosis are plotted in red. The largest blue (red) point shows the average location of individuals with nonsevere (advanced) fibrosis. The black triangles indicate the average location of individuals according to their mutation profile. Individuals with similar profiles are close to each other in the figure. For each continuous variable, the arrow provides a measure of the proportion of variance captured by a particular dimension. For example, dimension 1 captured approximately 75% of the variability of TGF-B1 and PDGF-BB.

Factor Analysis of Mixed Data (FAMD) consists of creating several dimensions, which are linear combinations of the original variables (here markers and type of mutations), to best explain the variance in the dataset. Individuals could then be visualized in the new space created by the first two dimensions (top of Fig. 3). The information shared between the continuous variables and the main dimensions is also shown (bottom of Fig. 3). The two main dimensions (x-axis = dimension 1, and y-axis = dimension 2) of the FAMD explained almost half of the variability in the data (46.7%). TGF-β1 and PDGF-BB (the most contributing variables to dimension 1 because these are the variables with the highest proportion of variability captured by this dimension) were found to be the variables that best explained the variability in serum levels between the wild type and the two other mutants. Otherwise, collagen and TIMP-1 (the continuous variables that contributed most to dimension 2) best explained the variability between BCP and PC mutation status. The overlap between individuals with nonsevere and advanced fibrosis is not very large showing that the markers and mutation profile are relevant to capture the differences between these individuals. Moreover, individuals with advanced fibrosis were in the lower plane, suggesting a protective effect of the double mutant with the simultaneous presence of the G1899A mutation against fibrosis, as previously described^17^.

#### Identification of factors associated with advanced fibrosis

**Figure 4:**
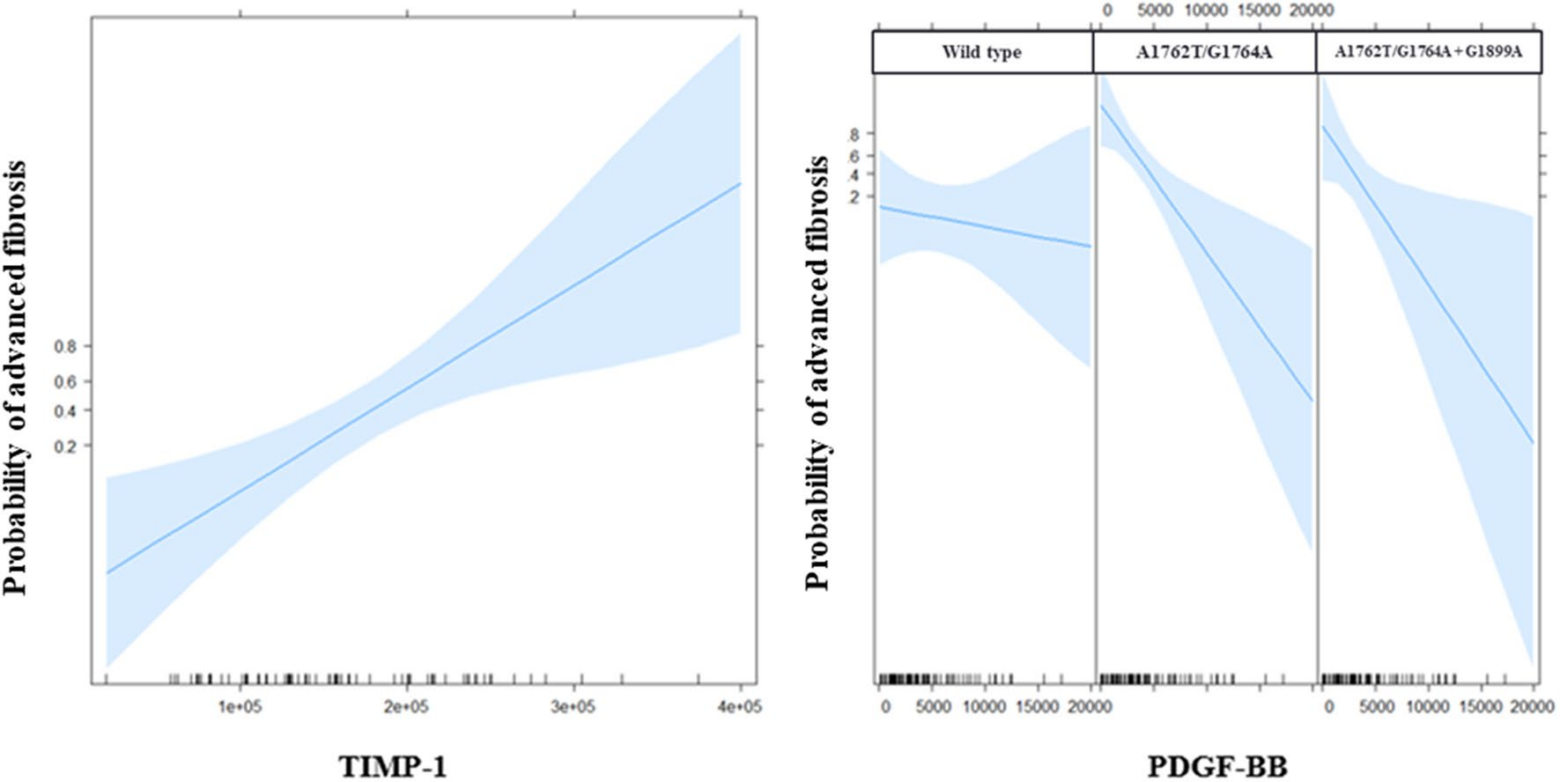
Effects of variables in the multivariate model of advanced fibrosis: TIMP-1 (left) and the interaction between PDGF-BB and the mutation profile (right) are on the X-axes; the probability of advanced fibrosis is on the Y-axes. The blue shades represent 95-percent pointwise confidence intervals around the estimated effects. The relationship between PDGF-BB and the probability of advanced fibrosis is steeper (slope of the curve) for individuals infected with viruses harboring BCP/PC mutations than for individuals infected with the wild type.

Binary logistic regression analysis in patients with fibrosis stage and BCP/PC mutation data showed that the serum levels of PDGF-BB (p value = 0.002) and TIMP-1 (p value = 0.001) and the mutation profile were independently associated with advanced fibrosis. Compared with individuals infected with virus harboring the A1762T/G1764A mutant, those infected with the wild type have a significantly lower risk of advanced fibrosis (p value = 0.003). A higher level of TIMP-1 was associated with advanced fibrosis regardless of the mutation status, and a higher level of PDGF-BB was associated with nonsevere fibrosis in patients infected with virus harboring the A1762T/G1764A mutant or both the double mutant and the G1899A mutation (Fig. 4).

### Correlations: marker serum levels according to BCP/PC mutants

**Figure 5:**
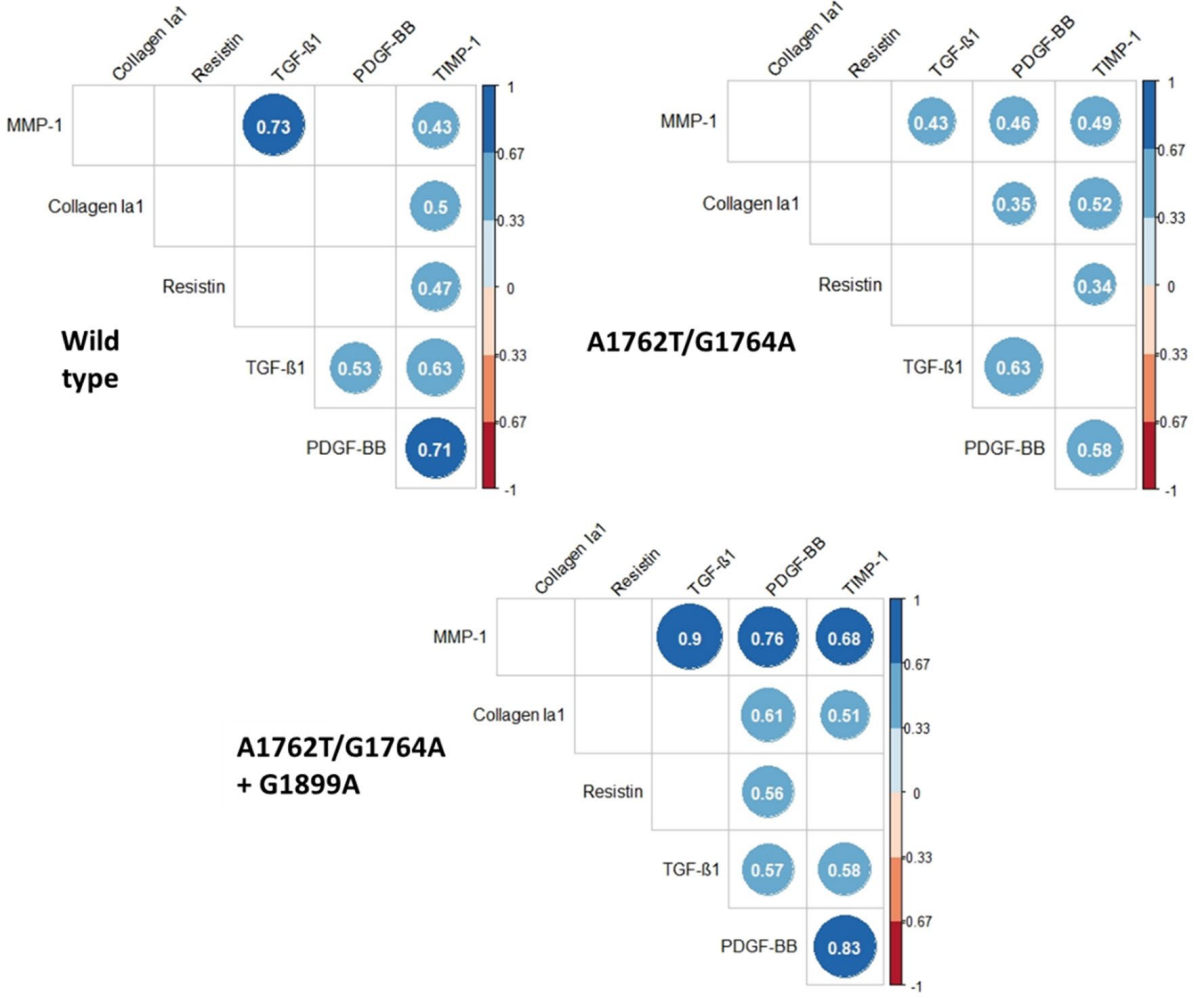
Correlations between the serum levels of liver markers (collagen IA1, resistin, TGF-β1, PDGF-BB, TIMP-1 and MMP-1) according to the mutation profile (wild type, A1762T/G1764A and A1762T/G1764A/G1899A). For each type of mutant profile, the pairwise Spearman correlation between the markers was calculated. These correlations are presented in the correlation matrix. When a correlation was not significant, the case was left blank (for example, the correlation between MMP-1 and Collagen IA1 was not significant for all profile types). Significant correlations are represented by discs with exact values of the Spearman correlation. Blue discs indicate positive correlations and red discs indicate negative correlations. The scale on the right side of the matrix indicates the range of the correlation. All exact values of the Spearman correlation coefficients are presented in the Supplementary data (Supplementary Table S3).

We assessed the correlations between the serum levels of liver markers according to the mutation profile (wild type, A1762T/G1764A and A1762T/G1764A/G1899A) (Fig. 5). TGF-β1 and PDGF-BB serum levels were significantly and positively correlated regardless of the mutation profile (mutated or not at nucleotide positions 1762/1764/1899). TGF-β1 and TIMP-1 serum levels were not correlated in the presence of the double mutant alone, but TGF-β1 and TIMP-1 levels were significantly and positively correlated for the wild type and the A1762T/G1764A/G1899A mutation profile. The significant and positive correlation between the serum levels of PDGF-BB and TIMP-1 was stronger for individuals infected with viruses harboring the wild type and the A1762T/G1764A/G1899A mutations than those infected with viruses harboring the double mutant only. These results show different correlation profiles depending upon the particular mutation profiles, suggesting an impact of the BCP/PC mutants (especially the A1762T/G1764A double mutant) on the secretion of marker serum levels.

## Discussion

HBV is not a directly cytopathogenic virus. The virus triggers an immune response from the host aimed at eliminating hepatocytes infected with HBV, resulting in the initiation of a dynamic process: fibrogenesis^23^. During the hepatic fibrogenesis process, HSCs undergo an activation process that leads to an imbalance in the synthesis and degradation of ECM. Thus, activated HSCs stimulate the production of MMP and lose of their primary function of storing vitamin A. Type IV and VI collagen fibers, present in the physiological state, are degraded by MMP enzymes and substituted predominantly by collagens I and III. Simultaneously, the production of TIMP-1 by the activated HSCs is also observed, reducing the hepatic fibrolytic process^23^. MMPs and TIMPs are therefore related to ECM fibrolytic processes^24^.

Liver fibrosis is the result of the interaction of multiple factors. In this study, we chose to analyze elements that are considered direct markers for measuring ECM degradation and fibrogenesis^25^. Indeed, we focused on the proteases involved in the degradation or modification of ECM (MMP-1 and TIMP-1), the products of ECM (collagen IA1) and fibrosis-related regulatory factors, such as PDGF-BB, TGF-β1, and resistin, all of whose essential roles in liver fibrosis have been established. Thus, previous studies showed that the levels of serum markers such as PDGF-BB, TIMPs and TGF-β1 were positively correlated with the degree of fibrosis, and their elevation suggests the activation of fibrogenesis^24,26,27^. In our study, the serum levels of these markers were analyzed according to the METAVIR scoring system. Nevertheless, the objective of the study was not to evaluate the analytical performance of these fibrosis markers in predicting liver fibrosis but to investigate the impact of BCP/PC mutants on the serum levels of these markers.

As previously described, our study confirms that a high serum level of TIMP-1 is associated with advanced fibrosis^28–30^. TIMP-1 is upregulated during hepatic fibrogenesis and is considered to promote fibrosis in the injured liver by inhibiting MMPs involved in the degradation of ECM. TIMP-1 therefore plays a pivotal role in the hepatic fibrolytic pathway. Even though the BCP double mutation is related to severe fibrosis, mutants in BCP or PC regions did not appear to impact the biological pathway involving TIMP-1. Previous research has demonstrated that the serum level of MMP-1 tends to decline with the severity of liver fibrosis, inflammation and the disease condition^28^. Our results, however, demonstrate that the serum level of MMP-1 was not significantly different between severe and nonsevere fibrosis stages.

Resistin is also upregulated under conditions of chronic injury in liver tissue and can act as an intrahepatic cytokine that can stimulate HSCs to secrete proinflammatory cytokines by activating the nuclear factor-κB signaling pathway^31^. Thus, serum resistin is involved in the pathophysiology of liver fibrosis and could serve as an indicator of disease severity in patients with hepatitis B (considering liver stiffness)^21^. In the present study, we confirm the previous findings, namely that the stage of severe fibrosis (considering in our study the METAVIR fibrosis score) is associated with a high level of resistin in the serum, but BCP/PC mutants did not impact the resistin serum level.

TGF-β is the most important cytokine in the progression of liver fibrosis because it is predominantly secreted by HSCs in response to hepatic injury and promotes the activation of HSCs. TGF-β1 is the predominant isoform of TGF-β among the three different isoforms identified in mammalian tissue. TGF-β1 can also participate in regulating ECM formation by decreasing and increasing the synthesis of proteases and the levels of protease inhibitors, respectively^32^. Additional growth factors, such as PDGF-BB become important in the later stage of HSC proliferation^28,33^ and therefore in the progression of liver fibrosis. Its biosynthesis is stimulated by TGF-β^34^. According to a number of studies, the serum level of TGF-β1 reflects the degree of hepatic fibrosis^35–38^, even if the predictive value of TGF-β1 for the diagnosis of fibrosis sometimes remains controversial^28,39^. Out of the three studies that discussed the clinical importance of PDGF-BB in the prediction of fibrosis, two studies were conducted among patients with CHB, and one study was among patients with chronic hepatitis C (CHC). In patients with CHB, Zhou et al. found a negative correlation between the fibrosis stages and serum PDGF-BB levels^40^, whereas two studies reported a positive correlation^27,28^. The study of patients with CHC identified that serum PDGF-BB levels were negatively correlated with fibrosis stages^41^, which is in agreement with the results described by Zhou et al. Our results show that the serum level of PDGF-BB is independently associated with advanced fibrosis. Nevertheless, a higher level of PDGF-BB seems to be associated with nonsevere fibrosis for patients infected by viruses harboring the A1762T/G1764A double mutant or both the A1762T/G1764A double mutant and the G1899A mutation. Likewise, TGF-β1 serum levels appeared to be higher in patients at a nonsevere stage of fibrosis than in patients at a severe stage. These results were not expected based on data previously reported in the literature, but those previous studies did not characterize the type of HBV mutant. Moreover, when we analyzed the correlation profiles between the serum levels of markers, we observed that TGF-β1 and TIMP-1 serum levels were not correlated in the presence of the double mutant alone. However, the TGF-β1 and TIMP-1 levels were significantly and positively correlated for the wild type and the double mutant/G1899A profile. These different correlation profiles between the serum levels of markers according to the mutation profile could suggest an impact of the double mutant on the secretion of marker serum levels. We can therefore assume that the mutation profile in the BCP/PC region (especially the A1762T/G1764A mutant) could interact with the biological process of fibrosis by directly or indirectly involving both TGF-β1 and PDGF-BB. This pathophysiological mechanism remains to be further elucidated.

This study has some limitations. Cytokine levels might be influenced by various factors such as comorbidities (obesity, hepatic steatosis and inflammatory diseases), immune system response, and platelet count. In HBV-infected patients with chronic diseases, blood cytokine levels may not be a reliable marker. They are not liver-specific and have a tend to be more elevated in the presence of inflammation, especially in the setting of liver fibrosis^24^ or when the body mass index increases^42,43^. The length of the evolution of chronic hepatitis may also have an impact on cytokine secretion levels, but such data are difficult to assess for consideration. The extrahepatic concentration of PDGF-BB would be related to the platelet count, and therefore, decreased serum levels of PDGF-BB could be explained by the decreasing platelet count during the progression of liver fibrosis in patients with CHB^44,45^. One of the weaknesses of the study is that we did not have access to platelet count data. The conflicting results observed between the studies may also be explained by differences in the immune system response to injury or the extent of liver damage in these patients. Furthermore, we did not use next-generation sequencing (NGS) for variant detection. The use of NGS could be interesting because NGS enables the characterization of viral variants with much greater sensitivity than is possible by standard population sequencing, as it detects variants at frequencies as low as 1% in the quasispecies pool^46^.

The concomitant impact of HBV genotypes on the secretion of markers would be interesting to investigate in a large cohort. Indeed, Neuman et al. investigated the impact of hepatitis C virus (HCV) genotypes and assessed differences in the progression of liver damage in individuals stratified by HCV genotypes. Thus, the HCV genotype is considered a variable that influences CHC progression, as it is for HBV^47,48^. Nevertheless, no significant difference has been observed in TGF-β levels according to HCV genotypes^47^.

This original study highlights the potential impact of the BCP double mutation on the serum secretion of markers involved in the fibrosis process. TGF-β1 and PDGF-BB are two essential growth factors in the development of liver fibrosis. The assessment of serum marker levels to reflect the fibrosis stage has been investigated in previous studies in HBV-infected patients, but these have not considered the possible impact of HBV mutants, including the BCP/PC mutants.

Our results suggest an impact of the A1762T/G1764A double mutant on biological processes related to TGF-β1 and PDGF-BB and provide additional data to support the potential role of BCP/PC mutants in the pathogenesis of liver injury in patients with chronic HBV infections. In vitro studies are needed to fully understand the impact of these mutants on the secretion of fibrosis markers and on the severity of liver fibrosis.

## Methods

### Patients

The samples were obtained from part of a national multicenter study (ANRS 2008-406, 14 participating virology laboratories) and a retrospective study including treatment-naive patients with chronic HBV infection^17^. The serum specimens were collected by the virology laboratory in Angers University Hospital between 2009 and 2013. The inclusion criteria were patients who had been HBV surface antigen (HBsAg)-positive for at least 6 months irrespective of the HBe status, with no prior or current anti-HBV treatment, with basal core promoter and precore mutation profiles available, and with all necessary laboratory/diagnostic data available (alanine aminotransferase [ALT], HBV DNA level, HBV genotype, or liver biopsy) as previously described^17^. All the included patients tested negative for hepatitis C virus, hepatitis Delta virus and HIV antibodies. This study was conducted in accordance with the principles of the Declaration of Helsinki. This study was approved by the Institutional Review Board of Angers University Hospital, and informed consent was obtained from all subjects^17^.

### Liver fibrosis

To assess liver fibrosis, all centers used liver biopsy^17^. The fibrosis stage was assessed according to the METAVIR scoring system, defined as follows: F0: no fibrosis; F1: portal fibrosis without septa; F2: portal fibrosis with rare septa; F3: numerous septa without cirrhosis; and F4: cirrhosis^49^. For the present study, the patients were classified as having severe (≥ F3) or nonsevere fibrosis (< F3). The choice of F3 stage as a cutoff was based on the results of a previous study that demonstrate that the F2 stage was critical to accurately evaluate the fibrosis level, as it was a source of variability in histological scoring^50^. The nonsevere fibrosis group was defined as the control group.

### Hepatitis B virus DNA quantification and genotyping

HBV DNA extraction from sera and real-time polymerase chain reaction (PCR) quantification were performed with a m2000sp/m2000rt automated system (Abbott Diagnostic, USA). The range of HBV quantification was 1 to 9 log IU/mL. HBV genotypes were determined by direct sequencing of the HBV polymerase gene, as described by Villeneuve et al.^51^.

### Detection of basal core promoter and precore mutations

The BCP (nucleotide position 1742–1849) and PC (nucleotide position 1814–1900) regions of the HBV genome were amplified by nested PCR as previously described^17,52^.

Nucleotide mutations were defined by their differences from the consensus sequence, and dual signals (mixed type) were considered a mutant type. The dominant viral strain was determined by Sanger sequencing and defined as > 50% of the virus.

### Fibrosis marker analysis

The secreted fibrosis markers were measured in the serum samples of patients by multiplex fluorescent-bead-based technology (Luminex 200, Austin, TX, USA) using two commercial Luminex screening assay kits: a customized Luminex Assay kit from R&D Systems (Lilles, France) for MMP-1, resistin, Collagen IA1, PDGF-BB and TIMP-1 and the Bio-Plex Pro TGF-β assay from Bio–Rad Laboratories (Marnes-la-Coquette, France).

In brief, the samples were diluted before incubation with specific antibody-coated fluorescent beads according to the manufacturer’s recommendations. After washing, 50 beads were analyzed with the Luminex 200™ analyzer and Bio-Plex Manager software version 6 (Bio–Rad Laboratories), and the analyte concentrations of the samples were estimated through the serial dilution of cytokine standards (MMP-1 sensitivity < 3 pg/mL; resistin sensitivity < 20 ng/mL; Col IA1 sensitivity < 100 pg/mL; PDGF-BB sensitivity < 50 pg/mL; TIMP-1 sensitivity < 800 pg/mL; and TGF-β1 sensitivity < 15 pg/mL).

### Statistical analysis

Quantitative variables are described as the means (standard error) or median (interquartile range) when appropriate. Categorical variables are described as total numbers (percentages).

Univariate analyses were performed to compare fibrosis markers in the serum samples according to fibrosis stage using Wilcoxon tests^53^ or according to the profile of mutation using Kruskal–Wallis tests^54^ and Steel–Dwass^55^ tests for post-hoc pairwise comparison. Proportions were compared using the chi-square test of independence. The data distributions were visualized in violin plots.

A first exploratory multivariate analysis was performed using FAMD, with the R package FactoMineR^56^ to analyze the variability of the data taking into account both the mutation profile and serum marker levels. Logistic regressions were then used to identify factors associated with advanced fibrosis. The candidate variables were MMP-1, resistin, Col IA1, PDGF-BB, TIMP-1, TGF- β1 and mutation profiles. The variables identified by univariate regressions (p value < 0.20) were then introduced in a multivariate analysis (stepwise backward logistic regression using Akaike Information Criterion for model selection). The regression formula used for this purpose also allowed us to identify factors independently associated with advanced fibrosis. Finally, we assessed the Spearman correlations between the serum levels of liver markers according to the mutation profile using the R package *corrplot*.

A p value lower than 0.05 was considered statistically significant. Statistical analyses were performed using R version 3.6.2.

## Supplementary Information

Supplementary Tables.

## Data Availability

The datasets generated during and/or analyzed during the current study are available from the corresponding author on reasonable request.
