# Supplementary material for: Analysis of hepatic fibrosis markers in the serum of chronic hepatitis B patients according to basal core promoter/precore mutants
Source: Sci Rep. 2022 Jun 17;12:10261. doi: 10.1038/s41598-022-14285-9 (PMC9205978; doi:10.1038/s41598-022-14285-9)
Supplement: Supplementary file 1 — Supplementary Tables. [file 41598_2022_14285_MOESM1_ESM.docx]

**Supplementary data**

**Title:** Analysis of hepatic fibrosis markers in the serum of chronic hepatitis B patients according to basal core promoter/precore mutants

**Authors:** Caroline Lefeuvre^1*^, Marine Roux^2^, Simon Blanchard^3^, Hélène Le Guillou-Guillemette^1^, Jérôme Boursier^1^, Françoise Lunel-Fabiani^1^, Pascale Jeannin^3^, Adeline Pivert^1^, Alexandra Ducancelle^1^

**Supplementary Table S1:** HBeAg and HBV DNA data according to fibrosis stage and mutation profile. The number of individuals does not allow performing a robust statistical analysis.

|  | | <F3 | | | ≥F3 | | |
| --- | --- | --- | --- | --- | --- | --- | --- |
|  |  | Wild type | A1762T/  G1764A | A1762T/G1764A + G1899A | Wild type | A1762T/  G1764A | A1762T/G1764A + G1899A |
| HBe Ag | Negative  Positive  Missing data | 10 (55.6%)  8 (44.4%)  1 | 16 (88.9%)  2 (11.1%)  2 | 8 (80.0%)  2 (20.0%)  2 | 1 (33.3%)  2 (66.7%) | 11 (57.9%)  8 (42.1%)  1 | 2 (100.0%)  0 (0.0%)  2 |
| HBV DNA (log IU/mL)  Missing data | | 4.6  [3.2; 9.5] | 4.0  [3.5; 5.1] | 4.1  [2.8; 6.1]  1 | 6.1  [5.4; 7.2] | 6.3  [5.1; 7.4] | 3.3  [2.9; 4.5]  1 |

<F3, nonsevere fibrosis (F0-F2); ≥F3, severe fibrosis (F3-F4); HBV, hepatitis B virus; HBeAg, hepatitis B e antigen.

**Supplementary Table S2:** The distribution of A1762T/G1764A mutants and A1762T/G1764A/G1899A mutants and severe/nonsevere fibrosis according to HBV genotype. The number of individuals does not allow performing a robust statistical analysis.

|  | HBV Genotype | | | | | | |
| --- | --- | --- | --- | --- | --- | --- | --- |
|  | **A** | **B** | **C** | **D** | **E** | **F** | **G** |
| Effective* | 36 | 2 | 9 | 13 | 12 | 4 | 1 |
| Severe fibrosis (≥F3) | 29 (81%) | 1 (50%) | 4 (44%) | 8 (62%) | 5 (42%) | 4 (100%) | 0 |
| Nonsevere fibrosis (<F3) | 7 (19%) | 1 (50%) | 5 (56%) | 5 (38%) | 7 (58%) | 0 | 1 (100%) |
| Wild type | 17 (47%) | 2 (100%) | 2 (22%) | 0 | 0 | 0 | 1 (100%) |
| A1762T/  G1764A | 14 (39%) | 0 | 7 (78%) | 8 (62%) | 8 (67%) | 2 (50%) | 0 |
| A1762T/  G1764A/ G1899A | 5 (14%) | 0 | 0 | 5 (38%) | 4 (33%) | 2 (50%) | 0 |

HBV, hepatitis B virus.

*2 missing data.

**Supplementary Table S3:** Exact values of Spearman correlation coefficients.

| **WILD TYPE** | | | | | |
| --- | --- | --- | --- | --- | --- |
|  | **Collagen Ia1** | **Resistin** | **TGF- β1** | **PDGF-BB** | **TIMP-1** |
| **MMP-1** | 0.038 | 0.023 | 0.733 | 0.234 | 0.426 |
| **Collagen Ia1** |  | 0.181 | 0.156 | 0.278 | 0.495 |
| **Resistin** |  |  | 0.364 | 0.350 | 0.469 |
| **TGF- β1** |  |  |  | 0.526 | 0.632 |
| **PDGF-BB** |  |  |  |  | 0.713 |

| **A1762T/G1764A** | | | | | |
| --- | --- | --- | --- | --- | --- |
|  | **Collagen Ia1** | **Resistin** | **TGF- β1** | **PDGF-BB** | **TIMP-1** |
| **MMP-1** | 0.268 | 0.211 | 0.433 | 0.458 | 0.485 |
| **Collagen Ia1** |  | 0.058 | 0.198 | 0.352 | 0.523 |
| **Resistin** |  |  | 0.044 | 0.062 | 0.344 |
| **TGF- β1** |  |  |  | 0.634 | 0.267 |
| **PDGF-BB** |  |  |  |  | 0.581 |

| **A1762T/G1764A/G1899A** | | | | | |
| --- | --- | --- | --- | --- | --- |
|  | **Collagen Ia1** | **Resistin** | **TGF- β1** | **PDGF-BB** | **TIMP-1** |
| **MMP-1** | 0.129 | 0.441 | 0.897 | 0.759 | 0.679 |
| **Collagen Ia1** |  | 0.288 | -0.032 | 0.606 | 0.509 |
| **Resistin** |  |  | 0.291 | 0.556 | 0.450 |
| **TGF- β1** |  |  |  | 0.574 | 0.579 |
| **PDGF-BB** |  |  |  |  | 0.829 |
